# Lack of a Sense of Threat and Higher Emotional Lability in Patients With Chronic Microvascular Ischemia as Measured by Non-linear EEG Parameters

**DOI:** 10.3389/fneur.2020.00122

**Published:** 2020-03-12

**Authors:** Galina V. Portnova

**Affiliations:** Human Higher Nervous Activity Lab, Institute of Higher Nervous Activity and Neurophysiology of RAS, Moscow, Russia

**Keywords:** EEG, chronic microvascular ischemia, emotional lability, fractal dimension, envelope, Hjorth

## Abstract

We aimed to investigate the non-linear features of the electroencephalogram (EEG) findings in patients with chronic microvascular ischemia (CMI) in order to determine the brain correlates of emotional impairment that could impact the risk of developing acute ischemia. We compared the EEG responses of patients with CMI and age-matched healthy volunteers to non-verbal emotionally charged sounds. We analyzed the EEG data, the psychological assessment of the stimuli, and the results of neuropsychological and behavioral testing. We assessed the (in)stability of the envelope's amplitude by calculating its average frequency and the ratio of its standard deviation to its mean. The non-linear features were lower in the patient group in the resting state. The emotional stimulation induced a decrease in the frequency of the envelope's amplitude in all subjects. Changes in the fractal dimension during stimulation were only seen in the patient group, and they correlated with symptoms of emotional lability. The lower ratio of the alpha-rhythm envelope's standard deviation to its mean in the right hemisphere correlated with a higher sense of threat. The EEG and behavioral correlates of emotional impairment in patients with CMI were found.

## Introduction

The impact of chronic microvascular ischemia (CMI) on cognitive function has garnered significant research interest in recent years (1), although the emotional impairment of patients with cerebral microvascular atrophy has not received a similar degree of attention. The emotional changes seen in this group of patients are significant due to the elevated risk of acute ischemia induced by emotional stress (2), as well as the reported increased incidence of underestimating the importance of medical treatment (3). The majority of data concerning emotional changes in patients with brain ischemia have been derived from post-stroke clinical cases. For example, it is well-known that depression is a typical complication stemming from a left-hemisphere stroke (4), which is associated with both disability (5) and worse rehabilitation outcomes in stroke survivors (6). Conversely, a right-hemisphere stroke is associated with the symptoms of anosognosia and euphoria (7). However, the emotional disturbances experienced by chronic ischemia patients who do not exhibit localized brain damage have only rarely been investigated. Some researchers have reported higher emotional lability and anosognosia in patients with vascular dementia (8). Our previous data showed that the electroencephalogram (EEG) resting state of patients with severe CMI was similar to that of those patients who had experienced a right-hemisphere stroke (9). In other studies, chronic ischemic changes in the white matter shown on the brain magnetic resonance imaging (MRI) had been associated with both cognitive and mood disturbances (10). Hence, we assumed that the emotional impairment of patients with chronic ischemia could barely be detected, which suggested that special methods of EEG analysis should be used. In particular, non-linear EEG parameters have previously been shown to be related to emotional states and affective reactions. For example, the fractal dimension of EEG signals provides comparative data concerning facial (11) and auditory (12) emotion recognition. The differential entropy of EEG signals has been proven to be more suitable for assessing emotion recognition than the traditional frequency domain EEG features (13). Further, the Hjorth parameters can be used for happiness/sadness recognition in relation to visual stimuli (14) and the emotional components of music (15). These non-linear features have been successfully used in clinical studies involving patients with emotional disturbances. For example, Pezard et al. (16) reported that depressive subjects tend to display lower complexity than control subjects. Moreover, it has been reported that decreased complexity and mobility are associated with insomniac subjects (17). Studies concerning post-traumatic stress disorder and panic disorder have reported lower Hjorth complexity in patients in pathological states when compared to healthy subjects (18).

The non-linear features of the EEG signal have also been shown to differ in patients with neurological disorders, such as epilepsy, attention deficit hyperactivity disorder (ADHD), and Alzheimer's disease (19). Therefore, we expected that the complexity and chaotic nature of the EEG data could prove useful for discriminating CMI. As reported by Mohammadi et al. (20), quantitative measures of chaos and non-linear features can be used to characterize the electrophysiological abnormalities seen in neuropsychiatric disorders that are not evident via linear analysis. The fractal dimension decreases during the acute phase of a stroke, which has also been associated with worse clinical recovery (21). Other studies have revealed that spectral entropy reflects the slowing down of brain activity seen in patients with post-stroke vascular dementia and stroke-related cognitive impairment, while permutation entropy and Tsallis entropy reflect the complexity of the examined signals (22). Additionally, it has been shown that non-linear complexity features can serve as a useful indicator when evaluating the stroke rehabilitation effect (23). Moreover, non-linear entropy analysis can be used to detect both the extent and the location of focal ischemic cerebral injury (24). The parameters of the EEG's envelope also showed a high specificity of EEG changes in patients after left-hemisphere stroke: the envelope's frequency was significantly lower in wide-band diapason (1.6–30 Hz). At the same time, the amplitude and frequency dynamics of the EEG envelope were sensitive to emotional changes of patients with a mental and cognitive deficit and the absence of local brain damage (25). Thus, the non-linear parameters of the EEG could potentially be used to detect the ischemic changes and the dynamics of brain activity that are correlated with cognitive processes and other brain functions, particularly emotional perception.

Therefore, the present study aimed to investigate the specific emotional response of patients with severe CMI that resulted in the atrophy of the cerebral hemisphere and cognitive deficit. We hypothesized that, when the cognitive impairment could be detected by neuropsychological and behavioral testing (26) and was previously associated with different EEG findings, the emotional-associated EEG changes could be hardly detected. In this point, the variable non-linear EEG features, which included Hjorth complexity, fractal dimension, and amplitude and frequency dynamics of the EEG's envelope, were applied to detect specificity of emotional impairment in patients with CMI. To identify any emotional changes, we presented the emotional auditory stimuli and registered the EEG changes using the non-linear features of the EEG.

## Materials and Methods

### Participants

Thirty-two patients with CMI and 45 age-matched healthy volunteers participated in the study (Table 1). All subjects were right-handed. Patients were recruited through the Hospital of RAS and Multidisciplinary clinic of Moscow. The MRI showed that patients had bilateral white matter lesions (Grades 2–3 by the Fazekas scale).

**Table 1:** Descriptive statistics of subjects.

**Groups**	**Number of subjects**	**Age**	**Sex (f/m)**	**IADL**	**TCD stenosis level (%)**	**Education level (years)**
Control group	45	65.6 ± 7.1	13/22	7.9 ± 0.1	10.9 ± 2.5	8.5 ± 1.3
CMI	32	67.9 ± 5.1	17/15	5.2 ± 0.6	59.7 ± 6.8	8.3 ± 1.2

*Mean ± SD*.

None of the participants had any prior psychiatric, neurological disorders or head trauma and were not taking any psychiatric medication at the time of the study. No participants took any anticonvulsants, nootropics, or antianxiolytics. This study was approved by the Ethics Committee of Institute of Higher Nervous Activity and Neurophysiology of RAS and Hospital of RAS. All patients and healthy volunteers provided their written informed consent after receiving a complete description of this study.

The study was conducted from 2015 to 2019. To analyze the correlates of the risk of acute ischemia for the future research, we performed regular outpatient examinations every 3–6 months after the study. MRI study aimed to confirm that the diagnosis of CMI was made every 1–2 years.

The statistics for worsening of CMI or developing acute ischemia (left hemisphere stroke) are presented in Table S1.

We analyzed hearing function in healthy subjects and patients as we presented auditory stimulation. We used a PDD-401 audiometer (Piston Ltd., Budapest, Hungary) to identify hearing threshold levels. None of the examined subjects had any symptoms of hearing loss.

### Clinical Assessment of the Level of Ischemia and Stroke Localization

Diagnoses of CMI were validated with a confirmation from CT or MRI performed by senior radiologists and was accompanied by multiple foci in the hemispheres indicating the developed brain atrophy. Fazekas scale was used to evaluate the severity of white matter lesions (27). Examination of the intracranial vessels was performed with color TCD with linear and phased probes at 2.1–2.5 MHz for each patient in the supine position after a 10-min rest. TCD showed signs of stenosis and, less frequently, occlusion of the extracranial arteries (stenosis level varied from 30 to 80%) in all patients. In healthy subjects, the degree of atherosclerosis of the arteries was <20%. Thus, all our patients had both macro- and microangiopathy.

### Neuropsychological Testing

#### Measuring the Time Interval During Attention Task

The participants were asked to find digits in ascending order using Schulte table (5 × 5 table with randomly distributed numbers were used) as fast as possible. This procedure was repeated twice.

#### Blind Clock Test

In this test, 10 pictures of clocks with no numbers were presented, one picture at a time, and the subjects were asked to tell what time was shown on the clock. We measured the number of corrected answers. This task utilized stimuli from the neuropsychological test booklet derived from Luria's battery (28, 29).

#### Verbal Memory Test

The list of five words were used for assessment of verbal short memory; the words in the set were selected and matched according to both frequency and length of words in Russian (30). We valuated total number of trials until the participant repeated all the words correctly, correctly recalled words after interpolation (subjects were asked to name sharp objects), the number of perseverative errors (separately confabulations and contaminations), and the order mistakes.

#### Visual Memory Test

An original test from Luria's battery [reprinted in Khomskaya (28)] was used (28, 29). We valuated total number of trials until the participant repeated all the figures correctly, correctly recalled figures after interpolation (subjects were asked to draw human), the number of the order, and spatial mistakes.

### Behavioral Assessments

Additional aspects of behavior were assessed using the Instrumental Activities of Daily Living (IADL) Scale (31).

### Stimuli

We presented 30-s-long sounds as stimuli. There was initially a large list (about 40) of such sounds, then a group of 198 healthy experts (mean age about 30 years) assessed all the stimuli on scales of pleasantness, arousal, sense of threat, gladness, etc. We selected seven stimuli for this study—most emotionally charged with most stable assessment: a dog barking, a crying infant, vomiting, coughing, scratching nails on glass, a bird singing, and human laughter. Resting states with open and closed eyes were recorded for 2 min in the beginning and in the end of the study. The subjects assessed emotional valence and arousal level of the stimuli by the scales pleasantness (from −5 to 5), arousal (from 0 to 10), fear (from 0 to 10), sense of threat (from 0 to 10), empathy (from 0 to 10), irritation (from 0 to 10), suspiciousness (from 0 to 10), and anxiety (from 0 to 10). The stimuli were presented in a random order for 30 s (8–10 times each) with 0.7- to 2.0-s gaps between them; the whole EEG experiment lasted about 50 min.

### EEG Recordings

During EEG recording, the subjects sat in a comfortable position after instruction to remain calm, with their eyes closed, to avoid falling asleep, and to avoid thinking about anything in particular. We recorded EEG (sampling rate of 250 Hz) using the EEG amplifier “Encephalan” (Medicom MTD, Taganrog, Russian Federation) with 19 AgCl electrodes placed according to the International 10–20 System. The electrodes placed on the left and right mastoids served as joint references under unipolar montage. The vertical electrooculogram (EOG) was measured with AgCl cup electrodes placed 1 cm above and below the left eye canthus, the horizontal 5 EOG was measured with electrodes placed 1 cm lateral from the outer canthi of both eyes. The electrode impedances were <10 kΩ.

### Procedure

The study was conducted from 2015 to 2019. Patients were invited to participate in the study approximately at the same time of the day—around 11:00–12:00. The EEG recording was performed after neuropsychological testing (10–15 min) and filling out of the IADL questionnaire (up to 10 min). We recorded resting-state EEG with eyes closed and eyes opened (5–7 min), and then subjects were instructed to keep their eyes closed and listen to the sounds that were presented by headphones. After the stimulation, participants were asked to assess the each stimulus by scales. The whole study took no more than 1.5 h.

### EEG Pre-processing

Continuous EEG fragments corresponding to the experimental sessions (rest and stimulation) of each subject was cleaned from eye movements and blinks by the Medicom plugin using electrooculogram (EOG) data. Muscle artifacts were cut out through manual data inspection. The continuous EEG of each subject was filtered with band-pass filter 0.5–30 Hz.

### Data Analysis

#### Fractal Dimension (FD)

We conducted the calculations of the examined signal bandpass-filtered in the range of interest (1.6–30 Hz), and a Butterworth filter of order 12 was used. FD was evaluated using the Higuchi algorithm (32).

#### Envelope Mean Frequency (EMF)

**Figure 1:**
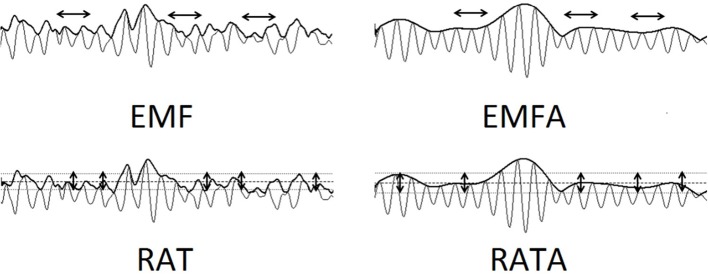
The visualization of Envelope's calculations: EMF—envelope mean frequency (1.6–30 Hz). EMFA—envelope mean frequency of the alpha-rhythm 8–13 Hz. RAT—ratio of its standard deviation to its mean (1.6–30 Hz). RATA—ratio of its standard deviation to its mean (8–13 Hz). EMF and EMFA reflect the frequency variability of the EEG data. RAT and RATA reflect the amplitude variability of the EEG data.

To express the (de-)synchronization dynamics of the rhythms, we applied the following method. First, we calculated the envelope of the EEG signal for the whole frequency range (1.6–30 Hz) and for the alpha rhythm (8–13 Hz) using the Hilbert transform (33). Second, we assessed the (in-)stability of the envelope's amplitude by calculating its average frequency using FFT (wideband—EMF, alpha—EMFA) and the ratio of its standard deviation to its mean (wideband—RAT, alpha—RATA, Figure 1).

#### Hjorth Parameters

Hjorth complexity (34) represents the change in frequency and indicates how the shape of a signal is similar to a pure sine wave. This parameter was calculated for wideband 1.6–30 Hz filtered signal in the following way: complexity (*y*(*t*)) =, where mobility (*y*(*t*)) =, *y*(*t*)—a signal, *y*′(*t*)—its derivative, and var(…)—the variance. The Hjorth complexity and mobility showed very similar results in our research, so we refer to them both as Hjorth parameters.

#### Power Spectral Density (PSD)

Additionally, the Fast Fourier transform was used to analyze PSD. The resulting normalized spectra were integrated over intervals of unit width in the range of interest (1.6–3, 3–4, … 29–30 Hz) in accordance with filtering of non-linear features.

### Statistical Analysis

A repeated-measures ANOVA with Bonferroni correction for multiple comparisons (*p* < 0.05) was used to determine group effects on EEG metrics (*post-hoc* Tukey). The group differences of assessment of stimuli were calculated using Mann–Whitney test. Only significant (*p* < 0.05) correlation values were used for further analysis.

### Cluster-Based Permutation Test

We analyzed a possible association of the EEG metrics with the ratings of neuropsychological and behavioral testing and subjective assessments of emotional stimuli using Spearman correlation analysis that was corrected for multiple comparisons by using clustering method (Matlab toolbox for BCI) with 500 permutations at each node (the Bonferroni-corrected *p*-value of 0.05). The permutation test was performed to compensate for the multiple statistical estimations of the correlations in different EEG channels. Correlation for each EEG channel was computed with Spearman correlation across subjects (35). Only significant (*p* < 0.05) correlation values were used for further analysis.

## Results

### Neuropsychological Testing and IADL: Group Differences and Correlations

The time of Schulte reading was significantly higher in the group of patients (*p* < 0.01); the parameters of verbal and visual memory also significantly differed between groups: the number of trials for correct recall was significantly higher and correctly recalled words/figures after interpolation were significantly lower in the group of patients. IADS scores differed significantly between groups (see Table 2).

**Table 2:** The results of neuropsychological testing.

	**Time of schulte reading**	**Verbal memory**		**Visual memory**		**Blind clock (number of corrected answers)**
		**Number of trials**	**Correctly recalled words after interpolation**	**Number of trials**	**Correctly recalled figures after interpolation**	
Patients	65.8 ± 7.9	3.1 ± 0.7	2.2 ± 0.5	3.6 ± 0.9	1.9 ± 0.7	34.6 ± 12.1%
Control group	39.1 ± 3.7	1.8 ± 0.5	4.6 ± 0.3	2.3 ± 0.6	4.1 ± 0.4	85.3 ± 9.4%

The IADL score positively correlated with difference of RATA between resting state and sounds of coughing and laughter (*r* = 0.495, *p* = 0.003) and sense of threat and suspiciousness (*r* = 0.521, *p* < 0.0007) for sounds of coughing, vomiting, barking, and laughter: the higher the RATA difference and sense of threat was, the higher IADL score the person had. The time of Schulte reading and number of trials required for correct recall of five figures negatively correlated with difference of RATA between resting state and sounds of coughing, barking, and laughter (*r* = −0.49, *p* = 0.002) and sense of threat (*r* = −0.524, *p* = 0.0008). We did not find other significant correlations between cognitive and behavioral assessment and EEG metrics or emotional assessment of stimuli.

### Emotional Assessment

The empathy level was significantly higher in the control group during presentation of coughing, vomiting, and crying (Mann–Whitney *U* test; *z* = 2.52, *p* = 0.008). The level of reported anxiety and sense of threat was significantly higher in the control group during presentation of coughing, vomiting, crying, laughter, and barking (Mann–Whitney *U* test; *z* = 2.67, *p* = 0.004). The arousal level was significantly higher (Mann–Whitney *U* test; *z* = 2.54, *p* = 0.008) in the control group during presentation of pleasant stimuli (bird singing and laughter) and was significantly lower during presentation of barking, coughing, and vomiting (Mann–Whitney *U* test; *z* = −2.37, *p* = 0.01) (Table 3).

**Table 3:** Subjective assessment of stimuli.

	**(0 10)**	**Bird singing**	**Coughing**	**Vomiting**	**Crying**	**Laughter**	**Barking**	**Scratching**
Control	Pleasantness	4.7 ± 0.2	−4.6 ± 0.9	−4.7 ± 0.7	−4.5 ± 0.3	4.1 ± 0.2	−4.6 ± 0.3	−4.5 ± 0.8
	Arousal	3.9 ± 0.5	6.8 ± 0.8	6.5 ± 0.9	6.8 ± 0.5	5.4 ± 1.2	6.0 ± 0.4	6.6 ± 0.7
	Empathy	1.3 ± 0.4	8.3 ± 1.1	8.5 ± 0.8	7.7 ± 0.9	7.9 ± 1.4	2.1 ± 0.9	0.8 ± 0.2
	Fear	0.6 ± 0.1	3.2 ± 0.9	2.9 ± 1.1	3.1 ± 0.6	1.2 ± 0.4	6.8 ± 1.2	4.1 ± 1.1
	Irritation	1.0 ± 0.4	3.2 ± 0.6	2.9 ± 0.8	3.0 ± 0.7	2.3 ± 0.5	4.3 ± 1.1	4.0 ± 0.9
	Anxiety	2.1 ± 0.5	7.8 ± 1.0	7.6 ± 1.2	5.4 ± 0.8	4.2 ± 1.0	6.6 ± 1.2	3.8 ± 0.9
	Suspiciousness	2.3 ± 0.6	6.5 ± 1.0	6.3 ± 0.8	3.8 ± 0.8	5.1 ± 1.1	4.9 ± 1.2	4.8 ± 0.7
	Sense of threat	3.1 ± 0.6	6.8 ± 1.0	6.6 ± 0.7	1.9 ± 0.9	5.5 ± 1.0	6.1 ± 0.9	2.0 ± 0.7
CMI	Pleasantness	6.9 ± 1.2	−7.4 ± 1.2	−7.8 ± 0.9	−3.0 ± 0.3	3.7 ± 0.9	−7.1 ± 1.1	−2.9 ± 1.1
	Arousal	1.1 ± 0.4	5.7 ± 0.9	5.1 ± 1.2	6.9 ± 0.9	3.6 ± 1.0	7.9 ± 0.7	4.7 ± 0.9
	Empathy	1.1 ± 0.6	3.9 ± 1.2	3.6 ± 0.9	3.0 ± 0.8	7.1 ± 0.7	1.1 ± 0.6	1.2 ± 0.6
	Fear	3.3 ± 0.7	3.0 ± 0.8	3.2 ± 1.3	2.2 ± 0.6	1.1 ± 0.6	6.8 ± 1.2	3.3 ± 1.0
	Irritation	5.0 ± 0.9	3.2 ± 1.6	3.0 ± 0.9	3.2 ± 0.9	2.9 ± 0.7	6.1 ± 1.1	3.4 ± 1.2
	Anxiety	2.3 ± 0.8	3.9 ± 1.0	4.1 ± 0.9	3.5 ± 0.8	2.0 ± 0.7	3.8 ± 0.9	2.1 ± 0.7
	Suspiciousness	1.8 ± 0.7	2.1 ± 0.8	2.0 ± 0.5	2.4 ± 0.8	1.5 ± 0.4	2.4 ± 0.7	1.8 ± 0.6
	Sense of threat	2.1 ± 0.8	2.8 ± 0.9	3.0 ± 0.7	3.2 ± 0.9	2.9 ± 0.7	3.2 ± 0.8	2.4 ± 0.5

*Mean ± SD*.

The sense of threat in healthy subjects negatively correlated with RATA. The data were corrected for multiple comparisons by cluster-based permutation test using clustering method with the Bonferroni-corrected *p*-value of 0.05. The significant negative clusters were found for sounds of coughing, barking, and laughter. The arousal positively correlated with FD in the frontal areas in both groups of subjects during listening of barking and coughing (Bonferroni-corrected *p*-value of 0.05) (**Figure 3A**). The Hjorth parameters negatively correlated with irritation in both groups of subjects in the frontal areas (**Figure 3B**). Significant permutation clusters were found for coughing and crying in the control group and for coughing in the group of patients (*p* < 0.05).

### Non-linear Parameters and PSD During Rest

**Figure 2:**
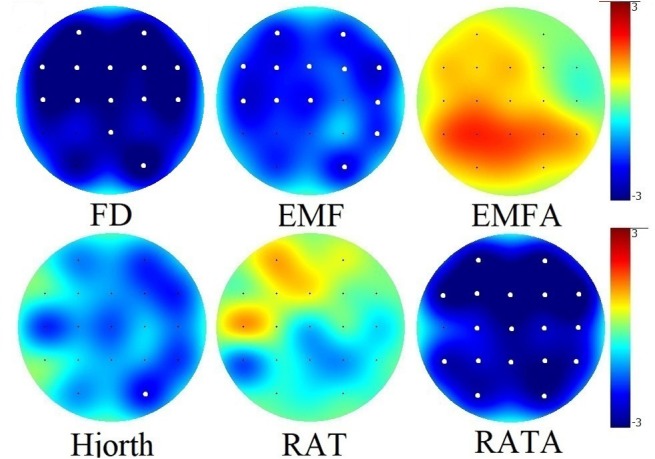
Group differences between values of non-linear EEG data during rest. Significant group differences after Bonferroni correction for multiple comparisons (*p* < 0.05) for each electrode are marked with a bold white dot.

The FD [*F*_(1, 75)_ = 17.468, *p* = 0.0008], EMF [*F*_(1, 75)_ = 10.795, *p* = 0.00155], and RATA [*F*_(1, 75)_ = 12.304, *p* = 0.0077] were significantly lower in the group of patients compared to controls during rest; the coefficients of significant difference are depicted in Figure 2. During the resting state, the group of patients had significantly higher 5–8 Hz PSD in frontal and central areas [*F*_(1, 75)_ = 24.975, *p* = 0.0001] compared to the control group.

### Non-linear Parameters and PSD During Emotional Stimulation

The group differences between the FD, EMF, and RATA parameters during stimulation were similar to the group differences during rest (Figure S1). These non-linear parameters were also lower in the group of patients (*p* < 0.05), except for FD and RATA during listening to the sounds of coughing or vomiting (Figure S1, Table 4).

**Table 4:** Values of non-linear parameters (averaged by all electrodes) during rest and stimulation (mean ± SD).

**Groups**	**Non-linear features**	**Rest**	**Bird singing**	**Coughing**	**Crying**	**Laughter**	**Barking**	**Scratching**
Patients with CMI	FD	1.26 ± 0.07	**1.21 ± 0.05**	**1.22 ± 0.04**	1.23 ± 0.05	1.27 ± 0.08	**1.22 ± 0.05**	1.24 ± 0.06
	EMF	4.40 ± 1.62	**2.84 ± 0.71**	3.40 ± 1.02	3.16 ± 0.82	4.49 ± 1.94	**2.72 ± 0.6**	3.42 ± 1.14
	EMFA	0.88 ± 0.11	0.66 ± 0.12	0.71 ± 0.15	0.67 ± 0.8	0.81 ± 1.16	0.61 ± 0.09	0.66 ± 0.09
	RAT	0.62 ± 0.09	0.62 ± 0.08	0.61 ± 0.09	0.59 ± 0.06	0.61 ± 0.09	0.61 ± 0.06	0.57 ± 0.09
	RATA	0.55 ± 0.08	0.56 ± 0.03	0.56 ± 0.06	0.55 ± 0.03	0.56 ± 0.04	0.56 ± 0.03	0.57 ± 0.05
	Hjorth Complexity	0.10 ± 0.03	0.09 ± 0.02	0.09 ± 0.02	0.09 ± 0.02	0.10 ± 0.03	0.09 ± 0.02	0.10 ± 0.03
Control group	FD	1.46 ± 1.01	1.36 ± 0.14	1.35 ± 0.08	1.47 ± 1.01	1.47 ± 1.02	1.37 ± 0.09	1.45 ± 0.09
	EMF	4.15 ± 1.89	4.38 ± 0.94	3.52 ± 1.19	4.41 ± 1.17	4.23 ± 2.05	4.99 ± 0.9	4.06 ± 1.39
	EMFA	0.58 ± 0.17	0.64 ± 0.19	0.54 ± 0.15	0.59 ± 0.13	0.57 ± 0.18	0.71 ± 0.12	0.57 ± 0.11
	RAT	0.58 ± 0.06	0.57 ± 0.04	0.56 ± 0.05	0.57 ± 0.05	0.57 ± 0.06	0.57 ± 0.07	0.57 ± 0.06
	RATA	0.65 ± 0.08	0.63 ± 0.07	**0.59 ± 0.05**	0.64 ± 0.07	0.62 ± 0.07	**0.60 ± 0.06**	0.63 ± 0.08
	Hjorth Complexity	0.10 ± 0.04	0.11 ± 0.03	0.11 ± 0.03	0.10 ± 0.05	0.10 ± 0.04	0.11 ± 0.03	0.10 ± 0.04

*Significant differences between stimuli and rest are marked by bold font*.

The effect of emotional stimulation differed in the control group and group of patients. The FD was significantly higher (only in group of patients) during presentation of barking [*F*_(1, 75)_ = 6.9956, *p* = 0.0997] and coughing [*F*_(1, 75)_ = 8.264, *p* = 0.0294] in the left frontal areas (Fp1, F7, F3, Fz, and T3) and was accompanied with higher arousal values of this stimuli. The sound of bird singing oppositely induced lower FD in the group of patients in the right parietal areas (F4, C4, Pz, and P4) [*F*_(1, 75)_ = 9.5571, *p* = 0.0280].

EMF was significantly low in all subjects during presentation of emotional stimuli compared to the resting-state condition [major stimuli effect *F*_(4, 144)_ = 2.8055, *p* = 0.1303]; the effect was most pronounced in the left hemisphere (O1, Pz, P3, T5, C3, Fz, T3, F3, and Fp1) in the group of patients [*F*_(1, 75)_ = 10.174, *p* = 0.0149] and in the left central and parietal areas (Pz, P3, P4, Cz, and C3) in the control group [*F*_(1,75)_ = 8.0833, *p* = 0.0575].

**Figure 3:**
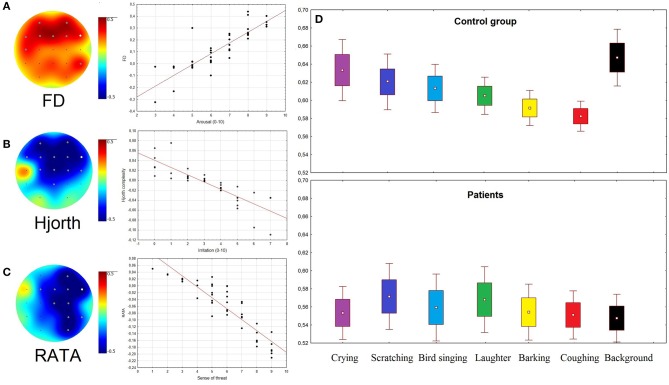
**(A–C)** Topographical plots showing the strength of Spearman correlation for all channels: **(A)** between Arousal rates and FD; **(B)** between Irritation rates and Hjorth complexity; **(C)** between rates of Sense of threat and RATA. Gray dots indicate channels with significant correlation (*p* < 0.05). Scatterplots of EEG patterns and ARSQ scores correspond to the channels marked as white dots. **(D)** RATA values in two groups of subjects during presentation of stimuli and rest (background condition).

The RATA parameter changed significantly only in healthy subjects during emotional stimulation in the right hemisphere (F8, F4, Fp2, C4, Cz, P4, and O2). It was significantly lower for coughing [*F*_(1, 75)_ = 23.087, *p* = 0.0001], barking [*F*_(1, 75)_ = 10.541, *p* = 0.0181], and laughter [*F*_(1, 75)_ = 8.7764, *p* = 0.0409] compared to background (Figure S1). The higher the sense of threat, the lower was the RATA values compared to background (Figures 3C,D).

The significant differences between PSD during stimulation and during rest were not registered. The 5–8 Hz PSD in frontal and central areas in the group of patients remained to be significantly higher for each type of stimuli compared to the control group (Figure S2).

## Discussion

In the present study, we compared the non-linear features of the EEG findings of patients with CMI and healthy controls, and we found significant differences both during emotional stimulation and at rest. In particular, the ratio of the alpha-rhythm envelope's standard deviation to its mean (RATA), the envelope's frequency (EMF), and the fractal dimension (FD) were lower in the patient group in the resting state. The other investigated non-linear EEG features did not differ between the groups. The parameters describe different sites of EEG variability, with the fractality, frequency, and amplitude being seen to be decreased in patients with chronic ischemia. These results match previous data regarding the reduced FD seen in patients with Alzheimer's disease (36), as well as the findings of prior studies reporting that spectral entropy reflects the slowing down of brain activity seen in post-stroke vascular dementia and stroke-related cognitive impairment patients (21). Other studies have reported that patients with cerebrovascular dementia exhibit the lowest coherence between the different cortical areas (37), while reduced alpha-rhythm amplitude has been noted in patients with chronic ischemia (38).

The emotional stimulation in the present study was accompanied by group-specific and non-specific changes in the EEG. The emotional response to auditory stimuli was not associated in our study with the dynamics of isolated frequency bands of PSD. These data were in accordance with our hypothesis that the non-linear features are more sensitive to emotional response compared to PSD as well as with some previous studies that reported that some emotional states could be better described by the cross-frequency amplitude dynamics of EEG but not the single alpha or theta band (39, 40). Moreover, the non-linear features, which previously showed an association with emotional response, correlated with different frequency bands (25). Regarding non-linear EEG features, EMF, which represents the dynamic changes in the EEG's frequency in the wide frequency range (1.6–30 Hz filtering), unlike EMFA (8–13 Hz), showed high sensitivity to the emotionally associated dynamics of EEG. The origin of the differences between EMF and EMFA could be associated with the high significance of slow-frequency and beta bands in the dynamics of envelopes' frequency according to the previous findings (41). So, the wide-band frequency range of filtering in the case of EEG's envelope calculations led to the significant decrease of EMF during the presentation of all the groups of stimuli, except for laughter and crying. This effect was found in all the subjects, and it did not correlate with any psychometric scores. Due to the absence of correlation with the psychometric scores, we assumed that the reported changes in the EMF could be explained by the perception of sounds, and hence, that they were not related to the emotional parameters of the stimulation. This assumption is consistent with the findings of previous studies, which showed that frequency changes in the EEG were associated with frequency changes in the EEG (42, 43). Further, auditory processing can alter the dynamics of ongoing rhythms of various frequencies, which may be related to perception, cognition, response selection, or a combination of processes (44).

Changes in the FD during stimulation were only found in the patient group, and they correlated with the arousal level during stimulation. In particular, during the bird song, which was accompanied by the lowest arousal level, the FD decreased, while during the laughter and barking (the highest arousal level), it increased. Some previous studies have reported that a higher emotional response is associated with a higher FD (45, 46). Furthermore, the FD was found to be useful in terms of detecting the level of arousal (47). In our previous study, we also observed a significant positive correlation between the FD of the EEG signal and the fMRI BOLD signal in certain regions, including the limbic system (48). Thus, the FD in the control group could be used as a measure of arousal, since it represents a higher level of emotional reactivity and lability than that seen in the patient group. Emotional lability and other mood and emotional changes are common syndromes following stroke and chronic ischemia (49–51). Moreover, patients with chronical vascular ischemia have been reported to exhibit even more severe emotional lability than stroke patients (52, 53). In our study, this phenomenon was found to be manifested in the higher fluctuations seen in the behavioral and EEG dynamics during stimuli perception, and it could be related to the lacunar damage to the white matter of the cortical–subcortical and frontal–parietal–occipital lobes visualized as multiple foci in the hemispheres of the MRI signal (54). Another explanation for the higher variability of the FD dynamics during stimulation could be related to the data showing that the patients with CMI had patchy and irregular brain damage (55), which indicates multiple small infarcts in the basal brain structures (56).

In the present study, the RATA, which could be interpreted as a measure of the alpha-rhythm amplitude instability, was found to be decreased in the right hemisphere, which was related to the emotional response to stimuli that induced a sense of threat. No prior studies have investigated changes in the RATA during emotional stimulation. However, earlier EEG-based studies of the alpha-rhythm's asymmetry have demonstrated that moderate fear activated the right hemisphere of the frontal lobes due to decreasing the amplitude and frequency of the alpha EEG waves (57). Other studies have reported that the right hemisphere is dominant for negative emotions (58), while reducing the excitability of the right frontal area by means of magnetic stimulation has been shown to reduce negative emotions (59).

From this perspective, the decrease of the RATA in the right hemisphere of the healthy subjects could be interpreted in light of the emotional response, which could be described as a sense of threat. In the patient group, the described EEG and emotional response was absent. This phenomenon could be explained by the symptoms of anosognosia (8) and cognitive impairment (60) seen in patients with microvascular ischemia.

The correlation between the cognitive impairment (i.e., the lower attention and visual memory), the low quality of life, and the low RATA found in our study supports previous findings (60). Hence, the lack of sense of threat or decrease of RATA in the group of patients during stimulation could be associated with their cognitive deficit or social disability.

So, our data demonstrated that some of the non-linear parameters could be used as markers of the developing CMI, as well as other EEG features did not show significant differences. In particular, the RATA was sensitive to both the emotional and cognitive impairment of patients with CMI; meanwhile, the dynamics of the RAT parameter were not significant. According to the previous investigations (25), both parameters corresponded to the variability of the EEG amplitude, and, according to some reports, unlike a wide-band filtering of EEG, the dynamics of alpha rhythms amplitude could be a sensitive marker of the intensity of emotion or arousal (61, 62). The rest of the non-linear EEG parameters did not correlate with any cognitive or behavioral ratings. At the same time, Hjorth's complexity, EMF, and FD were associated with emotional states. The dynamics of FD during emotional sounds were specific for patients with MCI and could be used to control the development of complications. In particular, the increase of FD associated with higher emotional reactivity of patients could potentially impact the risk of developing acute ischemia in this group of patients. According to this hypothesis, emotional hyperreactivity was previously associated with a higher risk of heart attacks or hypertensive crisis (63) and finally ischemic stroke (2, 64). Thus, the dynamics of FD associated with the emotional response in patients with CMI may have an impact on the risk of developing stroke and should be investigated further.

## Conclusions

In conclusion, the non-linear features (RATA, EMF, and FD) were found to be lower in the group of patients with chronic ischemia in the resting state. The significantly pronounced changes seen in the FD in response to emotionally charged stimulation in patients with CMI could be a sign of emotional lability. The decreasing RATA in the right hemisphere corresponded to a sense of threat in the healthy subjects. The patients with chronic vascular dementia demonstrated a lower sense of threat and less feelings of suspiciousness in response to emotional sounds, and they exhibited no decrease in the RATA.

## Limitations and Avenues for Future Research

The results of this study should be considered in the light of a number of limitations. First, the sample size is not large; second, in spite of homogeneity of MRI findings in the group of patients, we did not investigate the possible connection between the size of white matter lesions and EEG or behavioral data. Finally, according to the primary hypothesis, the EEG correlates of emotional impairment in patients with CMI may have an impact on the risk of developing acute stroke. To support our suggestions, we currently continue to collect longitudinal data about worsening of the disease or the development of stroke in our patients. The frequency range of non-linear parameters should be investigated further, especially for EMF and RATA indices.

## Data Availability Statement

The datasets generated for this study are available on request to the corresponding author.

## Ethics Statement

The studies involving human participants were reviewed and approved by the Ethics Committee of Institute of Higher Nervous Activity and Neurophysiology of RAS and Hospital of RAS. The patients/participants provided their written informed consent to participate in this study.

## Author Contributions

GP completed the study and prepared the article.

### Conflict of Interest

The author declares that the research was conducted in the absence of any commercial or financial relationships that could be construed as a potential conflict of interest.
