# Supplementary material for: Lack of a Sense of Threat and Higher Emotional Lability in Patients With Chronic Microvascular Ischemia as Measured by Non-linear EEG Parameters
Source: Front Neurol. 2020 Mar 12;11:122. doi: 10.3389/fneur.2020.00122 (PMC7080963; doi:10.3389/fneur.2020.00122)

**Portnova et al., Supplementary materials**

# **Lack of a sense of threat and higher emotional lability in patients with chronic microvascular ischemia as measured by non-linear EEG parameters**

**Table S1**. Mean scores and standard deviations of the evaluated ARSQ domains.

| N=32 | Year of participation | the year of ischemic stroke | the grade by the Fazekas scale | | Hypertensive emergencies per year | | arousal level (0-10) ** | FD difference between stimulation and rest *, ** |
| --- | --- | --- | --- | --- | --- | --- | --- | --- |
|  |  |  | MRI before study | MRI after study | before study | after study |  |  |
| P1 | 2015 |  | 2 | 2 | 2 | 1 | 6,0 | 0,033 |
| P2 | 2015 |  | 2-2 | 2-2 | 5 | 4 | 7,0 | 0,035 |
| P3 | 2016 | 2018 | 2 | 3 | 1 | 2 | 6,3 | 0,067 |
| P4 | 2016 |  | 2 | 2 | 0 | 0 | 4,3 | -0,012 |
| P5 | 2016 | 2017 | 2 | 2 | 3 | 2 | 6,3 | 0,059 |
| P6 | 2016 |  | 2 | 2 | 3 | 3 | 6,0 | -0,012 |
| P7 | 2016 |  | 2 | 2 | 0 | 0 | 6,0 | 0,005 |
| P8 | 2016 |  | 3 | 3 |  |  | 6,7 | 0,003 |
| P9 | 2016 |  | 2 | 2 | 0 | 0 | 5,3 | 0,005 |
| P10 | 2017 |  | 3 | 3 | 0 | 0 | 6,3 | 0,022 |
| P11 | 2017 |  | 2 | 2 | 0 | 1 | 5,3 | 0,016 |
| P12 | 2017 | 2018 | 2 | 2 | 1 | 1 | 8,3 | 0,122 |
| P13 | 2017 |  | 2 | 2 | 0 | 0 | 6,7 | 0,017 |
| P14 | 2017 |  | 3 | 3 | 1 | 0 | 7,3 | 0,021 |
| P15 | 2017 |  | 2 | 2 | 1 | 0 | 6,3 | 0,018 |
| P16 | 2017 |  | 3 | 3 |  | 1 | 7,0 | 0,030 |
| P17 | 2018 | 2018 | 3 | 3 | 4 | 2 | 7,3 | 0,039 |
| P18 | 2018 |  | 2 | 2 | 2 | 5 | 8,3 | 0,030 |
| P19 | 2018 |  | 2 | 2 | 2 | 0 | 7,0 | 0,025 |
| P20 | 2018 |  | 2 | 2 | 1 | 1 | 8,0 | 0,024 |
| P21 | 2018 |  | 2 | 2 | 0 |  | 8,3 | 0,295 |
| P22 | 2018 |  | 3 | 3 | 0 |  | 6,0 | 0,010 |
| P23 | 2018 |  | 2 | 2 | 0 |  | 6,7 | 0,017 |
| P24 | 2018 |  | 2 | 2 | 1 |  | 7,3 | 0,013 |
| P25 | 2018 |  | 3 | 3 | 1 |  | 8,3 | 0,027 |
| P26 | 2018 |  | 2 |  | 0 |  | 8,7 | 0,037 |
| P27 | 2018 |  | 2 | 2 | 1 |  | 7,3 | 0,050 |
| P28 | 2018 |  | 2 |  | 2 |  | 8,3 | 0,038 |
| P29 | 2018 |  | 2 |  | 3 |  | 7,7 | 0,042 |
| P30 | 2018 | 2019 | 3 | 3 | 2 |  | 9,0 | 0,071 |
| P31 | 2018 |  | 3 |  | 0 |  | 6,3 | 0,023 |
| P32 | 2019 |  | 2-3 |  | 0 |  | 5,0 | 0,004 |

| \| ** average of channels with significant differences* \| \| --- \| \| *** Average values of stimuli: barking, crying and coughing* \| |
| --- | --- | --- |

*Figure S1. Group differences between values of non-linear EEG during stimulation for FD, EMF and RATA. Significant group differences after Bonferroni correction for multiple comparisons (p < 0.05) for each electrode are marked with bold white dot.*

*
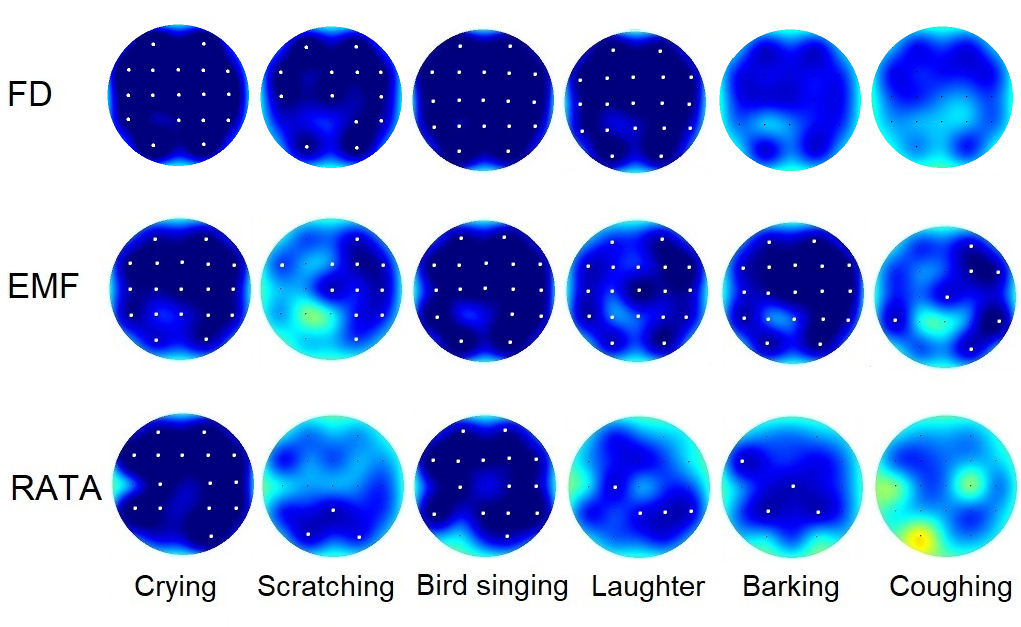
*

*Figure S2. Normalized PSD data during resting state (A) and stimulation (B).*


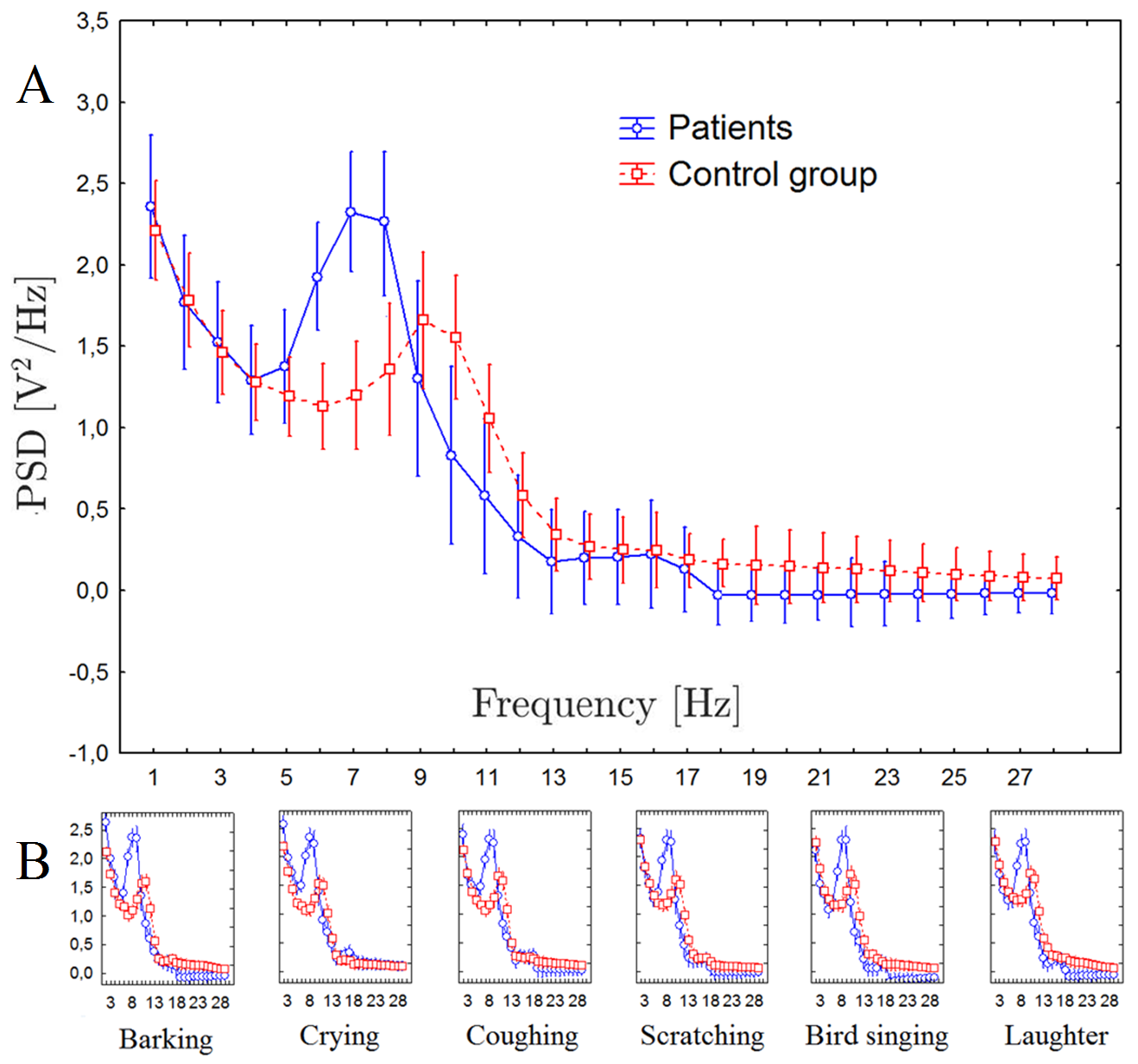

Supplement: Supplementary file 1 [file Table_1.DOCX]
